# Berberine is a Novel Mitochondrial Calcium Uniporter Inhibitor that Disrupts MCU‐EMRE Assembly

**DOI:** 10.1002/advs.202412311

**Published:** 2025-02-07

**Authors:** Haixin Zhao, Siqi Chen, Nian Cao, Wenjun Wu, Guangqin Liu, Jun Gao, Jiayi Chen, Ting Li, Dingyi Lu, Lingmin Zeng, Haizhen Zhu, Weina Zhang, Qing Xia, Teng Li, Tao Zhou, Xue‐Min Zhang, Ai‐Ling Li, Xin Pan

**Affiliations:** ^1^ Nanhu Laboratory National Center of Biomedical Analysis 27 Tai‐Ping Road Beijing 100039 China; ^2^ State Key Laboratory of Experimental Haematology Fifth Medical Center of Chinese PLA General Hospital Beijing 100071 China; ^3^ School of Basic Medical Sciences Fudan University Shanghai 200032 China; ^4^ Department of Cardiology the Sixth Medical Centre Chinese PLA General Hospital Beijing 100048 China

**Keywords:** berberine, EMRE, MCU (mitochondrial calcium uniporter), mitochondrial calcium signaling, myocardial injury, small molecules

## Abstract

The mitochondrial calcium uniporter (MCU) complex mediates Ca^2+^ entry into mitochondria, which plays a crucial role in regulating cellular energy metabolism and apoptosis. Dysregulation of MCU is implicated in various diseases, such as neurodegenerative disorders, cardiac diseases, and cancer. Despite its importance, developing specific and clinically viable MCU inhibitors is challenging. Here, Berberine, a well‐established drug with a documented safety profile, is identified as a potent MCU inhibitor through a virtual screening of an FDA‐approved drug library. Berberine localizes within mitochondria and directly binds to the juxtamembrane loop domain of MCU. This binding disrupts the interaction of MCU with its essential regulator, EMRE, thereby inhibiting rapid Ca^2+^ entry into the mitochondria. Notably, Berberine pretreatment reduces mitochondrial Ca^2+^ overload and mitigates ischemia/reperfusion‐induced myocardial injury in mice. These findings establish Berberine as a potent MCU inhibitor, offering a safe therapeutic strategy for diseases associated with dysregulated mitochondrial calcium homeostasis.

## Graphical abstract

Berberine, a safe and FDA‐approved drug, is identified as a novel mitochondrial calcium uniporter (MCU) inhibitor. By disrupting MCU‐EMRE interaction, Berberine prevents mitochondrial calcium overload and protects against ischemia/reperfusion injury, offering a promising therapeutic strategy for diseases linked to mitochondrial calcium dysregulation.

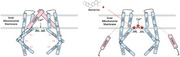

## 1 Introduction

The entry of Ca^2+^ ions into mitochondria primarily occurs through a highly selective Ca^2+^ channel known as mitochondrial Ca^2+^ uniporter (MCU).^[^ ^1^ ^]^ This process is crucial for regulating various cellular processes, including mitochondrial dynamics, energy metabolism, and cell death.^[^ ^2^ ^]^ The MCU complex, along with its regulatory components MICU1, MICU2, and EMRE, ensures precise control of mitochondrial Ca^2+^ uptake, which is crucial for maintaining cellular homeostasis.^[^ ^3^ ^]^ However, under pathological conditions, dysregulation of mitochondrial Ca^2+^ homeostasis may lead to detrimental effects. Excessive Ca^2+^ accumulation in mitochondria, referred to as mitochondrial Ca^2+^ overload, triggers mitochondrial permeability transition pore opening, leading to mitochondrial membrane depolarization, release of pro‐apoptotic factors, and ultimately cell death. These processes contribute to various diseases, including neurodegenerative disorders, cardiac diseases, and tissue damage caused by ischemia/reperfusion (I/R) injury.^[^ ^4^ ^]^

Despite the critical role of MCU in disease pathophysiology, developing effective therapeutic interventions targeting mitochondrial Ca^2+^ regulation remains challenging. Existing MCU inhibitors, such as ruthenium red and Ru360, exhibit poor delivery and toxicity issues.^[^ ^5^ ^]^ More recent inhibitors, including DS16570511^[^ ^6^ ^]^ and mitoxantrone,^[^ ^7^ ^]^ have shown promise in preclinical studies but are hampered by toxicity, limiting their clinical applicability. Ru265, a structural analog of Ru360, has shown improved cellular permeability, reduced cytotoxicity, and more efficient MCU inhibition in preclinical models.^[^ ^8^ ^]^ However, concerns regarding its off‐target effects, including seizure‐like behaviors, limit its broader use.^[^ ^9^ ^]^ These challenges underscore the need for next‐generation inhibitors that can overcome the limitations of existing compounds to provide a safer and more effective approach to targeting mitochondrial Ca^2+^ dysregulation. To advance the development of clinically viable MCU inhibitors, several key criteria must be met: 1) efficient penetration of cellular and mitochondrial membranes to effectively inhibit mitochondrial Ca^2+^ uptake; 2) selective modulation of mitochondrial Ca^2+^ uptake without affecting cytosolic Ca^2+^ signaling, ensuring normal cellular functions; 3) preservation of mitochondrial membrane potential, which is vital for mitochondrial function; and 4) low toxicity at therapeutically relevant concentrations to ensure safety and minimal adverse effects.

In response to the demand for safe and effective MCU inhibitors, we employed virtual molecular docking followed by functional screening of an FDA‐approved drug library. This led to the identification of Berberine, a natural alkaloid with an established safety profile, as a novel MCU inhibitor. Berberine localizes to mitochondria, directly binds to MCU, and disrupts the assembly of the MCU‐EMRE complex. Importantly, Berberine significantly reduces mitochondrial Ca^2+^ overload, providing cardioprotection against I/R‐induced myocardial injury in mice. This discovery positions Berberine as a promising candidate for further development in the broader context of diseases involving mitochondrial Ca^2+^ dysregulation.

## 2 Results

### 2.1 Screening for Potential MCU Inhibitors from an FDA‐Approved Drug Library

**Figure 1:**
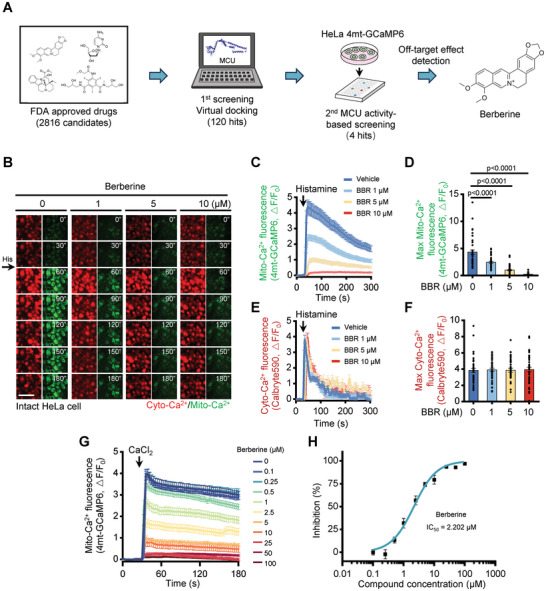
The discovery of Berberine as a novel MCU inhibitor. A) Schematic of screening workflow. B) Snapshot of cytosolic (left, red) and mitochondrial (right, green) Ca^2+^ transient images from time‐lapse movies of representative HeLa cells stained with Calbryte590 and 4mt‐GCaMP6 following 10 µm Histamine (His) stimulation. Cells were pretreated with various concentrations of Berberine. The time displayed on the images is in seconds. Scale bar, 50 µm. C) Representative traces of mitochondrial Ca^2+^ level (indicated by 4mt‐GCaMP6) in HeLa cells stimulated with 10 µm Histamine and pretreated with different concentrations of Berberine. Data are shown as mean ± s.e.m. *n* = 40 cells for each condition. D) Measurement of the maximal amplitudes of the mitochondrial Ca^2+^ traces in (C). Data are shown as the mean ± s.e.m. *n* = 40 cells for each condition. *P* value was analyzed using one‐way ANOVA. E) Representative traces of cytosolic Ca^2+^ level (indicated by Calbryte590) in HeLa cells stimulated with 10 µm Histamine and pretreated with different concentrations of Berberine. Data are shown as the mean ± s.e.m. *n *= 40 cells for each condition. F) Measurement of the maximal amplitudes of the mitochondrial Ca^2+^ traces in (E). Data are shown as the mean ± s.e.m. *n* = 40 cells for each condition. The *P*‐value was analyzed using one‐way ANOVA. G) Traces of mitochondrial Ca^2+^ level (indicated by fluorescence of 4mt‐GCaMP6) in digitonin‐permeabilized HeLa cells upon the addition of Ca^2+^ under varying concentrations of Berberine. H) Dose‐dependent inhibition of mitochondrial Ca^2+^ uptake activity by Berberine with an IC_50_ of 2.202 µm.

MCU functions as a major gate of Ca^2+^ flux from the cytosol into the mitochondrial matrix. Targeting MCU may serve as a therapeutic strategy for many diseases, such as pathological cardiac cell death.^[^ ^10^ ^]^ To closely emulate MCU‐targeted clinical treatment, we employed a screening approach using an FDA‐approved drug library comprising 2816 compounds (**Figure** **1**A). In our initial step, we performed a virtual screening via monomeric MCU structure‐based molecular docking. Based on the docking scores, we selected the top 120 compounds for further investigation, suggesting their potential MCU binding capacity (Figure S1A, Supporting Information). To assess the impact of these hits on mitochondrial Ca^2+^ uptake, we established HeLa cells expressing 4mt‐GCaMP6, a fluorescent indicator reflecting mitochondrial Ca^2+^ levels.^[^ ^11^ ^]^ Subsequently, we generated a screening system for mitochondrial Ca^2+^ uptake activity using permeabilized Hela cells. Exposure to 100 µm CaCl_2_ led to a rapid increase of the fluorescent signal, indicating mitochondrial Ca^2+^ uptake (Figure S1B, Supporting Information). Ru360, a well‐known MCU inhibitor,^[^ ^5^ ^]^ effectively blocked Ca^2+^ entry into mitochondria (Figure S1B,C, Supporting Information). In a small‐scale screen using the 120 hits, four small molecules demonstrated at least 50% inhibition of mitochondrial Ca^2+^ uptake at a concentration of 10 µm (Figure S1B–D, Supporting Information). Among them, Berberine exhibited the highest inhibitory effect, reaching up to 87% (Figure S1D, Supporting Information). To rule out the possibility that the observed effect was a secondary consequence of mitochondrial membrane potential disruption, the major driving force for Ca^2+^ uptake within the organelle,^[^ ^12^ ^]^ we performed a TMRM staining analysis. The mitochondrial decoupler, CCCP, induced a decrease in TMRM intensity. All tested drugs, except for Berberine, including Trifarotene, Eltrombopag, and Tipranavir, significantly decreased the mitochondrial membrane potential (Figure S1E, Supporting Information). Therefore, this screening process identified Berberine, a natural compound derived from medicinal herbs, as a promising candidate for further investigation as an MCU inhibitor.

### 2.2 Berberine Inhibits Mitochondrial Ca^2+^ Uptake

To assess Berberine's ability to traverse cellular membranes and inhibit mitochondrial Ca^2+^ uptake, we evaluated its inhibitory effects in intact cells by initiating intracellular Ca^2+^ signaling with histamine stimulation while simultaneously monitoring cytosolic and mitochondrial Ca^2+^ dynamics (Figure 1B). Berberine specifically inhibited mitochondrial Ca^2+^ uptake, as indicated by the green fluorescent indicator 4mt‐GCaMP6 (Figure 1C,D), without affecting the histamine‐induced cytosolic Ca^2+^ signals, as evidenced by the red fluorescent dye Calbryte590 (Figure 1E,F). Similar results were obtained using other Ca^2+^ indicators, cyto‐GCaMP6, or 4mt‐GCaMP8 in HeLa cells (Figure S2A–D, Supporting Information). In addition to pure berberine, its derivatives, including berberine hydrochloride and berberine sulfate, which enhance berberine's solubility,^[^ ^13^ ^]^ exhibited nearly identical inhibitory effects on mitochondrial Ca^2+^ uptake (Figure S2E,F, Supporting Information). These findings suggest that berberine and its derivatives can freely traverse the cellular membrane, thereby inhibiting mitochondrial Ca^2+^ uptake without impacting the cytosolic Ca^2+^ signals.

Next, to further quantify berberine's inhibitory effect on mitochondrial Ca^2+^ uptake, we permeabilized the cells with digitonin, allowing equal doses of calcium ions to be added outside the mitochondrial membrane, thus evaluating the capacity of mitochondria to absorb Ca^2+^ at different concentrations of Berberine. Berberine exhibited a potent inhibition of mitochondrial Ca^2+^ uptake in a dose‐dependent manner (Figure 1G), with an IC_50_ value determined to be 2.202 µm (Figure 1H).

To evaluate the safety of Berberine in cells, we treated the cells with increasing amounts of Berberine and confirmed that up to 10 µm Berberine treatment did not affect mitochondrial membrane potential by TMRM staining (Figure S2G,H, Supporting Information). We further investigated the cytotoxicity of Berberine to understand its potential impact on cell growth. It showed that at lower doses, specifically between 5–10 µm, which are sufficient to inhibit MCU, Berberine exhibited minimal cytotoxic effects (Figure S2I, Supporting Information). This indicates that Berberine is an effective MCU inhibitor with low cytotoxicity, making it a relative safe option at these concentrations.

### 2.3 Direct Binding of Berberine to MCU

Molecular docking screening indicates that BBR may directly bind to the MCU monomer (Figure 1A; Figure S1A, Supporting Information). This leads us to suggest that Berberine possesses the unique ability to directly traverse cellular membranes, enter mitochondria to target and subsequently inhibit MCU. Previous studies have documented the subcellular localization of Berberine within mitochondria.^[^ ^14^ ^]^ Exploiting Berberine's autofluorescence property, we also observed its cellular penetration at 10 µm, co‐localizing with MitoTracker DeepRed stain (Figure S3A,B, Supporting Information), reinforcing our hypothesis.

**Figure 2:**
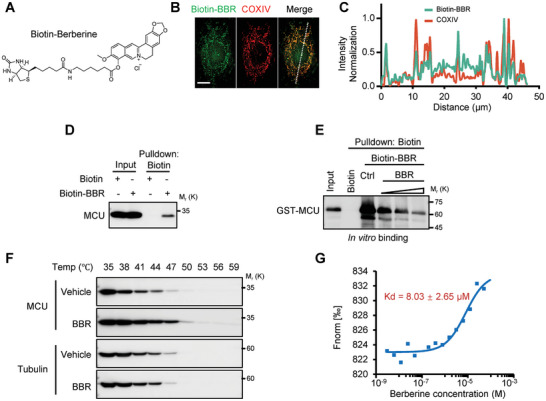
Berberine directly binds to MCU. A) Chemical structure of Biotin‐Berberine. B) Representative immunofluorescent images of HeLa cells treated with Biotin‐Berberine (green) and stained for mitochondria (COXIV, red). Scale bar, 10 µm. C) Traces of normalized fluorescence intensity spatial profiles through the white line shown in (B). D) Western blot analysis from Biotin‐Berberine pulldown assay using HEK293T cell lysates. Cells were treated with Biotin‐Berberine (10 µm, 2 h). E) Western Blot analysis from in vitro pulldown assay investigating interactions between GST‐MCU and Biotin‐Berberine under varying concentrations of Berberine. F) The cell lysates of HEK293T were incubated with or without Berberine for 2 h and then treated with increasing melting temperature (35 to 59 °C). The expression of MCU and Tubulin was detected by immunoblotting. G) Binding ability of Berberine to GST‐MCU in MST assays.

To further validate our hypothesis, we synthesized biotin‐conjugated Berberine (Biotin‐BBR) based on previous studies (**Figure** **2**A; Figure S4A–C, Supporting Information).^[^ ^15^ ^]^ The Biotin‐BBR exhibited similar mitochondrial localization (Figure 2B,C) and inhibition efficacy as unmodified Berberine (Figure S5A,B, Supporting Information), suggesting that the Biotin‐BBR retains the ability of interacting with the same molecular targets as the unmodified Berberine. Consistent with these results, Biotin pull‐down assays demonstrated that Biotin‐BBR could interact with both exogenous and endogenous MCU in cell lysate (Figure 2D; Figure S5C, Supporting Information). In vitro pull‐down assays further confirmed that Biotin‐BBR directly binds to recombinant glutathione‐S‐transferase (GST)‐MCU (Figure 2E). Notably, this binding was diminished when unlabeled Berberine competitively interacted with GST‐MCU, indicating a consistent binding site and confirming the specificity of the binding (Figure 2E).

To demonstrate the affinity of unmodified Berberine for MCU, we conducted a cell thermal shift assay. This assay is based on the principle that proteins tend to denature and precipitate upon heating, while ligand‐bound proteins often exhibit increased thermal stability.^[^ ^16^ ^]^ Our results showed that Berberine significantly enhanced the thermal stability of the MCU compared to the vehicle; meanwhile, Berberine did not affect the thermal stability of tubulin (Figure 2F), indicating that Berberine's interaction with MCU is relatively specific. Microscale thermophoresis (MST) analysis further confirmed that the affinity between GST‐MCU and Berberine was ≈8µm (Figure 2G).

### 2.4 Berberine Targets the Juxtamembrane Loop Domain of MCU

**Figure 3:**
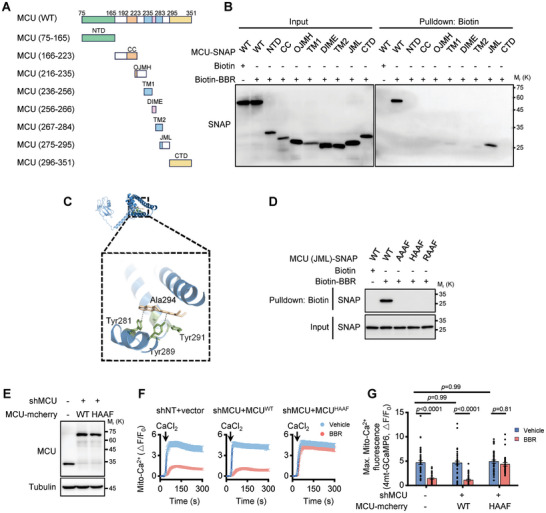
Berberine targets the JML domain of MCU to inhibit Ca^2+^ uptake. A) Schematic representation of MCU truncation mutants utilized for domain‐mapping studies. Numerical values in parentheses correspond to the amino acid sequences encompassed by each construct. Key structural domains are labeled: NTD (N‐terminal domain), CC (coiled‐coil domain), OJMH (outer juxtamembrane helix), TM (transmembrane helix), DIME (DIME motif, Ca^2+^ filter), JML (juxtamembrane loop), and CTD (C‐terminal domain). B) Western blot analysis of Biotin‐Berberine pulldown assays using in vitro transcribed and translated SNAP‐tagged MCU truncation mutants as shown in (A). C) Molecular docking model revealing the interaction between human MCU and Berberine. D) Western blot analysis of biotin‐berberine pulldown assays using in vitro transcribed and translated SNAP‐tagged MCU mutants (amino acids 275–295, encompassing the JML region). Wild‐type residues Y281, Y289, Y291, and A294 were mutated to AAAF, HAAF, and RAAF, respectively. E) Western blot analysis of endogenous MCU and exogenous MCU‐mCherry expression in HeLa stable cell lines with MCU wild‐type (WT) and MCU^HAAF^ mutants. F) Traces of mitochondrial Ca^2+^ levels, as indicated by the fluorescence intensity of 4mt‐GCaMP6, in digitonin‐permeabilized HeLa cells following Ca^2+^ addition. The cells include control HeLa, and stable cell lines expressing mCherry‐tagged MCU^WT^and MCU^HAAF^ mutants, under conditions with and without 10 µm Berberine. G) Measurement of the maximal amplitudes of the mitochondrial Ca^2+^ traces in (F). Data are shown as the mean ± s.e.m. *n* = 45 cells for each condition. The *P*‐value was analyzed using two‐way ANOVA.

Next, to explore the mechanism of Berberine's binding to MCU and its functional impact, we first mapped MCU regions interacting with Berberine. We in vitro translated MCU truncations based on its secondary structure,^[^ ^17^ ^]^ excluding the mitochondrial targeting sequence (residues 1–74) due to its non‐conservative nature.^[^ ^18^ ^]^ We assessed their interactions with Berberine using Biotin pull‐down assays. Berberine showed a strong affinity for the MCU juxtamembrane loop (JML) segment spanning residues 275–295 (**Figure** **3**A,B), although minor binding to other areas was also observed (Figure 3B). These observations were consistent with molecular docking simulations that examined the interactions between Berberine (or biotin‐Berberine) and MCU (Figure 3C; Figure S5D,E, Supporting Information). The simulations indicated that Berberine (or biotin‐Berberine) binds near the JML domain, a region critical for EMRE‐dependent MCU opening.^[^ ^17^, ^19^ ^]^ Docking analyses pinpointed key binding residues, including Tyr 281, Tyr 289, Tyr 291, and Ala 294 (Figure 3C; Figure S5D,E, Supporting Information). Notably, Tyr 281 and Tyr 289 are likely pivotal for hydrogen bonds with Berberine as shown by molecular docking simulations (Figure 3C; Figure S5D, Supporting Information).

To explore the interaction of Berberine with predicted binding sites within the MCU, we performed site‐directed mutagenesis. Sequence alignment of the JML domain revealed four conserved residues (Y281, Y289, Y291, and A294) across various MCU homologs (Figure S5F, Supporting Information), known to play a role in maintaining the open conformation of the MCU.^[^ ^19^ ^]^ It was reported that mutations of Y289 and Y291 to alanine, as well as A294 to phenylalanine, do not alter the open conformation of MCU and preserve robust mitochondrial Ca^2+^ uptake capacity. However, the critical residue Y281, when mutated to alanine, induces a closed conformation of the MCU.^[^ ^19^ ^]^ Under these circumstances, simultaneous mutations of Y281A, Y289A, Y291A, and A294F significantly disrupted the interaction with the Berberine‐MCU JML domain (Figure 3D), confirming Berberine's direct binding to these residues. To determine whether the ability of Berberine to bind was independent of the MCU's conformational state (open or closed), we introduced mutations Y281H and Y281R, which maintain the open conformation^[^ ^19^ ^]^ but disrupt potential hydrogen bonding with Berberine. These mutations still abolished Berberine binding (Figure 3D), negating the hypothesis that Berberine's binding is confined to the open conformation of MCU.

To further demonstrate that Berberine's inhibition of the calcium ion absorption function of the MCU is dependent on its binding to the JML domain, we engineered cell lines with stable reintroduction of either the wild‐type MCU (MCU^WT^) or the MCU^HAAF^ mutant (Figure 3E). This experimental design enabled us to contrast the effects of Berberine on mitochondrial Ca^2+^ uptake across these scenarios. Berberine treatment led to a reduction in mitochondrial Ca^2+^ uptake in both control and MCU^WT^‐reconstituted cells (Figure 3F,G). In contrast, cells expressing the MCU^HAAF^ mutant, which does not bind Berberine due to alterations in the JML domain (Figure 3D), exhibited no decrease in mitochondrial Ca^2+^ uptake following Berberine treatment (Figure 3F,G). This divergence underscores the critical role of the JML domain interaction in Berberine's mechanism of action on mitochondrial Ca^2+^ regulation.

### 2.5 Berberine Disrupts MCU‐EMRE Interaction

**Figure 4:**
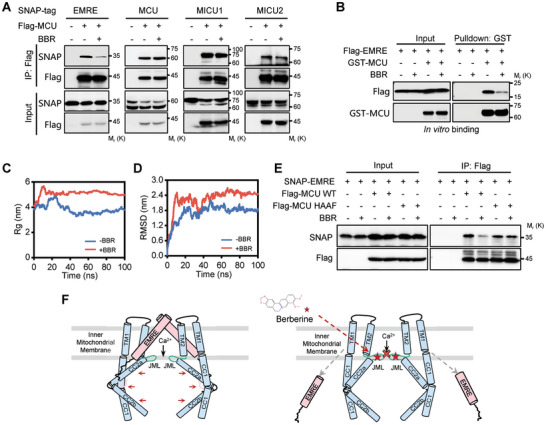
Berberine inhibits Ca^2+^ uptake by disrupting MCU‐EMRE complex assembly. A) Western blot analysis depicting the co‐immunoprecipitation of Flag‐tagged MCU with SNAP‐tagged EMRE, MCU, MICU1, and MICU2 in HEK293T cells, conducted with or without the presence of 10 µm Berberine for 2 hours. B) Western blot analysis of GST pulldown assays performed with in vitro transcribed and translated Flag‐tagged EMRE and recombinant GST‐MCU, with and without the addition of 2 µg Berberine. C) Molecular dynamics simulations showing the radius of gyration (*R*g) of the MCU‐EMRE complex in the presence and absence of Berberine. D) Molecular dynamics simulations depicting the root‐mean‐square deviation (RMSD) of the MCU‐EMRE complex with and without Berberine. E) Western blot analysis of the co‐precipitation of Flag‐tagged MCU wild‐type (WT) or Flag‐tagged MCU^HAAF^ mutant with SNAP‐EMRE in HEK293T cells, treated with 10 µm Berberine for 2 h or untreated. F) Proposed model for Berberine‐mediated MCU gating. Left panel, EMRE binds to the MCU and stabilizes the luminal gate of the MCU in the open conformation. Right panel, Berberine integrates into the MCU, inducing a closed conformation that disrupts the assembly of the MCU‐EMRE complex and seals the MCU's luminal gate.

Ca^2+^ transfer through the mitochondrial inner membrane is mediated by the mitochondrial calcium uniporter (MCU) complex, consisting of MCU, EMRE, MICU1, and MICU2.^[^ ^3^ ^]^ We investigated Berberine's influence on the MCU complex assembly via co‐immunoprecipitation (IP) experiments. The components of the MCU complex, including MCU, EMRE, MICU1, and MICU2, were SNAP‐tagged and co‐expressed with Flag‐tagged MCU in 293T cells. Following Berberine treatment, only the interaction between SNAP‐tagged EMRE and Flag‐tagged MCU was significantly diminished, while the interactions of MCU with other MCU components remained unaltered (**Figure** **4**A). These results suggest that Berberine selectively disrupts the EMRE‐MCU interaction, which was further confirmed by a reverse IP assay (Figure S6A, Supporting Information).

To directly assess Berberine's effect on MCU‐EMRE interaction, we employed in vitro binding assays. These assays confirmed Berberine's ability to directly interfere with MCU‐EMRE assembly (Figure 4B). To understand the mechanism behind this disruption, we conducted molecular dynamics simulations. The simulations revealed that the MCU‐EMRE complex rapidly reached equilibrium and maintained stability during the simulation in the absence of Berberine. At 100 ns, the *R*_g_ value, a measure of compactness, significantly increased from 3.734 to 4.982 nm upon Berberine addition (Figure 4C), indicating a more spread‐out and less compact complex structure. This observation was further corroborated by the increased RMSD values (reflecting average atomic displacement) in the presence of Berberine (Figure 4D). Together, these results suggest that Berberine induces a looser and less stable complex structure (Figure S6B, Supporting Information). Furthermore, hydrogen bond analysis revealed a reduction in the average number of bonds between MCU and EMRE from 10.540 to 6.287 upon Berberine treatment (Figure S6C,D, Supporting Information), signifying a weakening of interactions between the two proteins. This finding aligns with the significant decrease in binding energy observed between MCU and EMRE in the presence of Berberine (from −100.02 ± 17.85 to −29.52 ± 27.07 kcal mol^−1^, Figure S6E, Supporting Information). Collectively, these data strongly support the conclusion that Berberine disrupts the MCU‐EMRE complex by weakening the interactions between MCU and EMRE.

Next, we sought to clarify whether Berberine's disruption of the MCU‐EMRE binding activity depends on its interaction with the MCU JML domain. We co‐transfected cells with MCU^WT^ or the Berberine (BBR)‐binding deficient mutant MCU^HAAF^ alongside EMRE. Both MCU^WT^ and MCU^HAAF^ were capable of interacting with EMRE, indicating that the mutation in the JML domain itself does not affect its assembly with EMRE (Figure 4E). The addition of Berberine disrupted the assembly between MCU^WT^ and EMRE; however, when Berberine could not bind to the MCU JML domain, as with MCU^HAAF^, Berberine's presence did not influence the MCU‐EMRE assembly. These findings, together with our molecular dynamics simulation results (Figure 4C,D; Figure S6B–E, Supporting Information), suggest that Berberine's modulation of mitochondrial Ca^2+^ uptake is contingent upon its interaction with MCU. This interaction subsequently disrupts the MCU‐EMRE complex assembly and ultimately leads to the inhibition of the MCU channel, thereby preventing Ca^2+^ entry into the mitochondria (Figure 4F).

### 2.6 Berberine Suppresses Mitochondrial Ca^2+^ Overload and Protects against Myocardial Ischemia‐Reperfusion Injury

**Figure 5:**
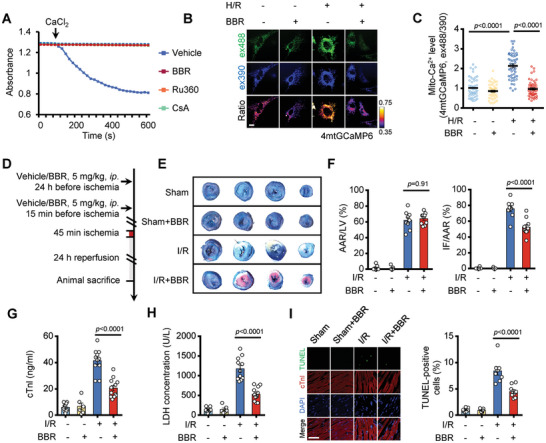
Berberine protects against myocardial ischemia/reperfusion injury through inhibiting mitochondrial Ca^2+^ overload. A) Mitochondrial swelling in response to Ca^2+^ challenge (500 µm CaCl_2_). Swelling is inhibited with Berberine (10 µm), Ru360 (3 µm), or CsA (3 µm). B) Representative images of neonatal rat cardiomyocytes expressing 4mt‐GCaMP6. Cells were either treated with 10 µm Berberine or not, then subjected to hypoxia (3 h) followed by reoxygenation (12 h) (H/R). Fluorescence at ex488 (green) represents the Ca^2+^‐dependent signal, while fluorescence at ex390 (blue) indicates the Ca^2+^‐independent signal. The ratio (pseudocolor) of ex488/ex390 indicates the level of mitochondrial Ca^2+^. Scale bar, 10 µm. C) Measurement of mitochondrial Ca^2+^ level indicated by 4mt‐GCaMP6 fluorescence ratio (ex488/ex405) in (B). Data are shown as the mean ± s.e.m. *n* = 50 cells for each condition. The *P*‐value was analyzed using one‐way ANOVA. D) The experimental scheme of the mice with acute I/R injury (45 min ischemia followed by 24 h reperfusion) with vehicle or Berberine pretreatment (24 h and 15 min before the onset of ischemia). E) Evans Blue and 2, 3, 5‐triphenyltetrazolium chloride (TTC) staining of myocardium after I/R injury. The blue area indicates a non‐ischemic area. The red indicates viable tissue at risk. The white indicates an infarct area. F) Quantification of infarct size (IF), area at risk (AAR) of mice subjected to acute I/R injury (as protocols in D). *n* = 9 or 10 mice. G–I). Cardiac cell death indexed by serum cTnI (G, *n* = 12 [sham], 6 [sham+BBR], 11 [I/R], and 12 [I/R+BBR]), serum LDH concentration (H, *n* = 13 [sham], 6 [sham+BBR], 11 [I/R], and 12 [I/R+BBR]), and myocardial TUNEL‐positive cells (I, *n* = 8 [sham], 6 [sham+BBR], 11 [I/R], and 12 [I/R+BBR]) of the mice subjected to I/R injury (as the protocols in D) with vehicle or Berberine treatment (5 mg kg^−1^, ip.24 h and 15 min before I/R injury). *P* value was analyzed using one‐way ANOVA. Scale bar, 50 µm.

We next sought to explore the physiological relevance of our findings. It's well established that excessive Ca^2+^ entry into mitochondria can lead to mitochondrial Ca^2+^ overload. This overload subsequently triggers the opening of the permeability transition pore (PTP), causing mitochondrial swelling, and eventually, cell death.^[^ ^20^ ^]^ Cardiac‐specific conditional knockout of MCU could protect mitochondria by preventing the opening of mPTP in vitro and in vivo.^[^ ^10a,b^ ^]^ The mPTP opening can be monitored by measuring mitochondrial absorbance.^[^ ^21^ ^]^ In an in vitro context, we observed that Berberine inhibited Ca^2+^ overload‐induced mitochondrial swelling (**Figure** **5**A). This effect paralleled those of Ru360 and cyclosporin A (CsA),^[^ ^22^ ^]^ a known inhibitor of mPTP opening, suggesting that Berberine might confer cellular protection under conditions of mitochondrial Ca^2+^ overload.

To validate this hypothesis, we first established a model of hypoxia/reoxygenation (H/R) injury in neonatal rat cardiomyocytes and embryonic rat cardiomyocyte‐derived H9c2 cell line. Consistent with previous studies,^[^ ^23^ ^]^ H/R injury led to a significantly elevated mitochondrial Ca^2+^ level, indicated by the fluorescent intensity of 4mt‐GCaMP6 or Rhod2 (Figure 5B,C; Figure S7A,B, Supporting Information). Furthermore, Berberine pretreatment alleviated this mitochondrial Ca^2+^ overload (Figure 5B,C; Figure S7A,B, Supporting Information). To assess the potential cardioprotective effect of Berberine in vivo, a mouse acute cardiac ischemia/reperfusion (I/R) model was established (Figure 5D). Pretreatment of Berberine (24 h plus 15 min before reperfusion) significantly reduced I/R‐induced myocardial infarct size (Figure 5E,F). Additionally, Berberine ameliorated cardiomyocyte death, evidenced by decreased cTNl and LDH concentration (Figure 5G,H). TUNEL staining experiments further confirmed the protective effect of Berberine against myocardial I/R injury (Figure 5I). In summary, these data indicate that Berberine exhibits therapeutic effects in myocardial I/R injury via counteracting mitochondrial Ca^2+^ overload.

## 3 Discussion

Our virtual screening of an FDA‐approved drug library identified Berberine, a natural alkaloid derived from medicinal plants, as a potent MCU inhibitor. Mechanistically, Berberine exerts its inhibitory effect by directly binding to the MCU and disrupting the assembly of the MCU‐EMRE complex, consequently hindering Ca^2+^ entry into the mitochondrial matrix. Our findings demonstrate that Berberine interacts with residues Y281, Y289, Y291, and A294, which are located near the JML domain of MCU. The JML is essential for channel gate formation and its interaction with EMRE is critical for the activation of the Ca^2+^ channel.^[^ ^17^ ^]^ Mutational analyses further underline the significance of Y281 and Y289 in the MCU‐EMRE assembly and channel opening.^[^ ^19^ ^]^ For example, the Y281A mutation severely disrupts EMRE binding,^[^ ^19^ ^]^ which corresponds to a loss of Ca^2+^ uptake similar to that observed upon Berberine treatment. The hydrophobic nature of Berberine^[^ ^24^ ^]^ likely promotes its integration near the JML, disrupting critical interactions like Y281 and Y289 and stabilizing a closed MCU conformation that prevents EMRE binding. Molecular dynamics simulations support this hypothesis, highlighting the JML as a promising target for small‐molecule modulation. However, further structural studies are necessary to fully clarify the mechanisms of MCU gating and the role of small molecules in this process.

Berberine exhibits potent inhibitory effects on mitochondrial Ca^2+^ uptake, coupled with a well‐established safety profile, supported by extensive clinical and preclinical studies.^[^ ^25^ ^]^ Unlike existing MCU inhibitors such as Ru360, Ru265,^[^ ^8^ ^]^ and DS16570511,^[^ ^6^ ^]^ which suffer from cytotoxicity or lack comprehensive toxicity evaluations, Berberine stands out for its proven safety in cellular, animal, and clinical studies.^[^ ^25^ ^]^ This makes it a compelling candidate for treating diseases associated with mitochondrial Ca^2+^ dysregulation. In contrast, Mitoxantrone, another FDA‐approved drug, inhibits topoisomerase II and PKC at concentrations ranging from nanomolar to low micromolar levels,^[^ ^26^ ^]^ which are lower than its MCU inhibitory concentration.^[^ ^7^ ^]^ This difference restricts its clinical application primarily to leukemia treatment, as its MCU inhibitory concentration is not achievable at therapeutic doses without significant toxicity. Berberine, on the other hand, is considered safe at therapeutic doses, making it a more versatile candidate for broader clinical applications. Clinical trials have reported minimal side effects, primarily transient gastrointestinal disturbances such as diarrhea and nausea at higher doses,^[^ ^25^, ^27^ ^]^ which are reversible upon discontinuation.^[^ ^28^ ^]^ While long‐term safety data for Berberine are limited, its ability to acutely modulate mitochondrial Ca^2+^ transients makes it a promising therapeutic agent for conditions involving mitochondrial Ca^2+^ overload.

Beyond its role in MCU inhibition, Berberine also possesses diverse pharmacological effects, such as anti‐inflammatory, antioxidant, and antidiabetic activities.^[^ ^29^ ^]^ These properties contribute to its broad clinical applications, such as the treatment of cardiovascular diseases, type 2 diabetes, gastrointestinal disorders, and bacterial infections.^[^ ^25^, ^28^, ^30^ ^]^ Notably, its antibacterial and antitumor effects are linked to its interaction with DNA, disrupting replication and transcription.^[^ ^31^ ^]^ In diabetes, Berberine enhances insulin secretion by targeting the KCNH6 potassium channel, thus improving glucose homeostasis.^[^ ^25a^ ^]^ In ischemia‐reperfusion (I/R) injury, Berberine has demonstrated significant cardioprotective effects, supported by clinical evidence showing its ability to modulate inflammatory markers in patients with acute myocardial infarction.^[^ ^32^ ^]^ While earlier studies attributed these effects to the maintenance of mitochondrial integrity and respiratory complex activity,^[^ ^33^ ^]^ the precise molecular targets remained unclear. Our findings establish MCU as a key cardiac target of Berberine, revealing a core mechanism for its protective effects in I/R injury. Given that mitochondrial Ca^2+^ overload occurs rapidly within the initial 20 min post‐ischemia,^[^ ^23b^ ^]^ early intervention with Berberine is of paramount importance.

In conclusion, our study identifies Berberine as a novel MCU inhibitor with significant potential for clinical applications. Revealing its inhibitory effects on MCU and providing insights into the complex Berberine‐MCU‐EMRE dynamics, our findings lay a solid foundation for the development of targeted drug strategies aimed at mitochondrial Ca^2+^ regulation. Structural analogs of Berberine could be engineered for enhanced specificity and safety as MCU inhibitors. Furthermore, the role of MCU dysfunction is becoming increasingly understood, not only in cardiac I/R injury but also for other diseases, such as the recently reported dependency of venetoclax‐resistant leukemic stem cells (LSCs) on mitochondrial calcium for survival.^[^ ^34^ ^]^ These findings position Berberine as a valuable starting point for developing novel interventions in diseases associated with dysregulated mitochondrial Ca^2+^ homeostasis.

## 4 Experimental Section

### Chemicals and Reagents

Berberine (#910263, purity: ≥98%) was purchased from J&K Scientific (Beijing, China). Ru360 (#557440‐1SET, purity: ≥95%) was obtained from Merck (Sigma‐Aldrich, St. Louis, MO, USA). Berberine chloride (#HY‐18258, purity: ≥98%), Berberine sulfate (#HY‐N0716B, purity: ≥98%), and the FDA‐approved drug library (HY‐L022) were sourced from MedChemExpress (Monmouth Junction, NJ, USA). Berberine, Berberine chloride, and Berberine sulfate were dissolved in water to prepare 1 mm stock solutions, with sonication required to ensure complete dissolution. Ru360 was dissolved in DMSO to prepare a 10 mm stock solution.

### Experimental Animals

All animals used in this study were maintained in the pathogen‐free barrier animal facility at the National Center of Biomedical Analysis. Animal care was monitored daily by certified veterinary staff and laboratory personnel. 10‐week‐old mice in the C57BL/6J background and pregnant Sprague–Dawley rats were purchased from the Beijing Vital River Laboratory Animal Technology Company. The experiments were conducted following approval by the Institutional Animal Care and Use Committee of the National Center of Biomedical Analysis (IACUC‐DWZX‐2020‐731).

### Murine Model of Myocardial Ischemia

The 10 week‐male‐C57BL/6 mice (>22 g) were randomly assigned to specific groups. Berberine (5 mg kg^−1^) or vehicle was administered intraperitoneally twice (24 h and 15 min prior) to ischemia‐reperfusion (I/R) surgery as described.^[^ ^35^ ^]^ Briefly, pentobarbital (70 mg kg^−1^ i.p.) was used to induce and maintain anesthesia. Mice were then ventilated on a Harvard rodent respirator via a tracheostomy. A midline sternotomy was performed, with a reversible snare occlude placed around the left anterior descending coronary artery. I/R injury was initiated by tightening the snare for 45 min, then releasing it. After reperfusion for 24 h, the mice were sacrificed for further detection and measurement.

For serum LDH concentration and cTnl level, blood samples were centrifuged at 3000 rpm for 10 min and then detected using an LDH kit (Sigma‐Aldrich, Cat# MAK066) and a mouse cardiac troponin I ELISA kit (ThermoFisher, Cat#EEL112) according to the manufacturer's instructions.

For the measurement of the area‐at‐risk (AAR) and infarct size, the heart was initially excised post‐reocclusion of the coronary artery at the previous occlusion site. The heart ascending aorta was cannulated (distal to the sinus of Valsalva) and then perfused retrogradely with 0.05% Alcian blue to delineate the AAR. Following AAR visualization, the heart was frozen at −80 °C for 5 min, cut into ≈1 mm slices, and the unstained infarcted (IF) region was lucifugal visualized using 1% 2,3,5‐triphenyl‐tetrazolium chloride at 37 °C for 15 min. Infarct and left ventricular areas were determined by planimetry with Image J. The infarct size was calculated as infarct area divided by area at risk (IF/AAR).

### Cell Culture

To establish HeLa cells stably expressing 4mt‐GCaMP6s, a lentiviral vector (pLVX‐puro) containing the 4mt‐GCaMP6s insert was constructed. This vector was transfected into HEK293T cells along with the packaging plasmids psPAX2 and pVSV‐G using a 4:3:1 ratio. Stable transfectants were selected using 1 µg ml^−1^ puromycin. HeLa cells were cultured at 37 °C in DMEM supplemented with 10% FBS and 1% penicillin‐streptomycin. A similar approach was employed to express MCU WT and MCU HAAF in the HeLa cell line. Initially, HeLa cells were infected with the pLKO.1‐shMCU lentivirus and subsequently selected with 1 µg ml^−1^ puromycin. Knockout HeLa cells were then infected with lentivirus‐carrying pLVX‐mCherry‐N1 vectors harboring either MCU^WT^ or MCU^HAAF^. Cells expressing mCherry were isolated using flow cytometry. Knockout of endogenous MCU and expression of exogenous MCU‐mCherry were confirmed by Western blot analysis. All other mammalian cell lines used in this study were from the American Type Culture Collection. All the cell lines were fully authenticated and tested free of mycoplasma.

### Mitochondrial Ca^2+^ Modulator Screening

Hela cells expressing 4mt‐GCaMP6s were permeabilized by a 1 min perfusion with intracellular buffer (130 mm KCl, 10 mm NaCl, 2 mm K_2_HPO_4_, 5 mm Succinic acid, 5 mm Malic acid, 1 mm Pyruvate, 0.5 mm ATP, 0.1 mm ADP, MgCl_2_, 20 mm HEPES, pH 7, 37 °C) supplemented with 10 µm digitonin and 50 µm EGTA. Then the buffer was changed to intracellular buffer supplemented with 10 µm compounds from primary screen hits of FDA‐approved drugs for 10 min. Mitochondrial Ca^2+^ uptake was evoked by adding 100 µm CaCl_2_ to the buffer. Ca^2+^ imaging was performed using a DeltaVision Deconvolution microscope (GE Healthcare) equipped with a ×20 objective. Exposure time was set to 25 ms for FITC. Images were acquired at 1 frame per 3 s with an EM‐CCD camera. Images were analyzed using Image J software. All images were background‐corrected by subtracting mean pixel values of a cell‐free region of interest.

### Molecular Docking Screening

To ensure unbiased docking results, the monomeric form of MCU was primarily used as the receptor for subsequent docking studies. The 3D structure of the MCU monomer (PDB: 6XJV) was obtained from the Protein Data Bank. A set of 2816 FDA‐approved drugs were converted to 3D structures using Open Babel. Preparation and parameterization of the receptor protein and ligands were conducted with AutoDock Tools (ADT3). Docking grid files were generated with AutoGrid, ensuring the docking box fully encompassed the MCU monomer. Docking simulations were then performed with AutoDock Vina (1.2.0).^[^ ^36^ ^]^ The score was ranked according to the value of the binding energy of the protein‐ligand complex. Finally, the protein‐ligand interaction diagrams were analyzed and visualized using Discovery Studio 2019 and PyMOL.

### Molecular Dynamics (MD) Simulations

Structural data for MCU and EMRE monomers were retrieved from the Protein Data Bank (PDB) via the entry code 6XJV. The preprocessing phase employed Gromacs 2018 as the simulation suite, coupled with the Amber99SB‐ildn force field for encompassing protein and small molecule interactions. A 10 × 10 × 10 nm^3^ water box, utilizing the TIP3P model, was constructed to solvate the system, ensuring a minimal 1.2 nm buffer between the protein surfaces and the box boundaries. This was followed by ion addition for electrostatic equilibrium.

For treating electrostatic interactions, the Particle‐mesh Ewald method was implemented. Energy minimization was executed through the steepest descent method over 50 000 steps, setting both the Coulombic and van der Waals interaction cutoffs at 1 nm. Equilibration phases under NVT (constant number of particles, volume, and temperature) and NPT (constant number of particles, pressure, and temperature) settings preceded a 100 ns MD simulation, conducted under standard laboratory conditions of temperature and pressure. Non‐bonded interactions were applied with a 10 Å cutoff. Thermal and pressure stability were maintained at 300 K and 1 bar, respectively, utilizing the V‐rescale thermostat and the Berendsen barostat.

This rigorous preparation and execution strategy yielded a robust framework for accurately simulating the complex's behavior in a physiological context, culminating in the acquisition of data such as radius of gyration (*R*g), root‐mean‐square deviation (RMSD), number of hydrogen bonds (H‐bonds), and total free energy, to assess structural stability, conformational dynamics, interaction strength, and energetics throughout the simulation period.

### Ca^2+^ Measurements

For imaging Ca^2+^ transients evoked by treatments with an agonist, cells labeled with Calbryte590 or 4mt‐GCaMP6 were incubated at 37 °C with Krebs–Ringer modified buffer (KRB), which contained 125 mm NaCl, 5 mm KCl, 1 mm Na_3_PO_4_, 1 mm MgSO_4_, 5.5 mm glucose and 20 mm HEPES (pH 7.4). After 10 µm histamine stimulation, a series of images were captured every 3 s using a DeltaVision Deconvolution microscope (GE Healthcare). The recorded images were analyzed and quantified using Image J software (NIH).

For image analysis, background correction was first performed frame by frame by subtracting the intensity of a nearby cell‐free region from the signal of the imaged cell. F_0_ was calculated as the initial background‐subtracted fluorescence intensity. F indicated the background‐subtracted fluorescence intensity at each time point. Δ*F*/*F*_0 _= (*F* − *F*_0_)/F_0_, indicated the Ca^2+^ concentration change after stimulation.

### Mitochondrial Membrane Potential Measurements

For confocal microscopy, adherent HeLa cells were stained with Tetramethyl‐rhodamine (TMRM) at a final concentration of 100 nm in a fresh culture medium. After a 30‐minute incubation period at 37 °C to allow for adequate dye uptake, cells were treated with the compounds of interest for an additional 30 min at the same temperature. Subsequently, cells were washed to remove excess dye and unbound compounds. Imaging was performed on a DeltaVision confocal microscope (GE Healthcare). Acquired images were then processed and quantified using ImageJ software.

For flow cytometric analysis, Hela cells were digested and stained with 100 nm Tetramethyl‐rhodamine (TMRM) in fresh medium for 30 min at 37 °C. Compounds were added for 30 min at 37 °C. Then cells were washed twice and resuspended in PBS containing 7AAD. Samples were analyzed using a cytoFLEX flow cytometer (Beckman Coulter).

### Western Blot Analysis

For western blot analysis, cells were lysed in RIPA buffer (50 mm Tris‐HCl (pH 8.0), 150 mm NaCl, 1% (v/v) Nonidet P‐40, 0.5% sodium deoxycholate, 0.1% SDS, protease inhibitor cocktail). Protein samples were resolved by SDS‐PAGE and transferred onto PVDF membranes. Membranes were immunoblotted with the indicated primary antibodies. Antibodies were used at the following concentrations: anti‐MCU, rabbit polyclonal, Cell Signaling Technology, Cat# 14997 (1:1000); anti‐SNAP, rabbit polyclonal, New England Biolabs, Cat# P9310S (1:1000).

### Immunoprecipitation

HEK293T cells were transfected with the indicated plasmids using Lipofectamine 2000 transfection reagent (ThermoFisher, Cat# 11668500). After transfection for 24 h, cells were treated with 10 µm Berberine for 2 h before harvested. Cells were lysed with RIPA buffer (50 mm Tris‐HCl (pH 8.0), 150 mm NaCl, 1% (v/v) Nonidet P‐40, 0.5% sodium deoxycholate, protease inhibitor cocktail).

Cell lysates were immunoprecipitated by anti‐Flag M2 affinity gel (Selleck, Cat# B23102) overnight at 4 °C. Beads were washed 5 times in RIPA buffer followed by eluting bound protein complexes in 2× SDS dye. Whole‐cell lysates and co‐precipitation samples were analyzed by western blot.

### Synthesis and Characterization of Biotin‐Berberine

Biotin‐Berberine was synthesized by Pharmaron following a previously reported protocol.^[^ ^15^ ^]^ Briefly, Berberrubine and 6‐biotin aminohexanoic acid were first prepared and then coupled in DMF using HATU to yield Biotin‐Berberine.

### Synthesis of Berberrubine

Berberine hydrochloride (1 g, 2.69 mmol) was heated at 195 °C under a nitrogen atmosphere for 20 min until the yellow solid turned dark red. After cooling to room temperature, 40 mL of a solvent mixture (ethanol: hydrochloric acid, 2:38 v/v) was added to acidify the reaction. The precipitate was collected by filtration, washed with ethanol (3 × 10 mL), and purified using reversed‐phase flash chromatography (C18 silica gel column, MeCN in water with 0.05% HCl, gradient 5% to 80% over 30 min, UV detection at 254 nm). The purified fraction was lyophilized to yield Berberrubine (450 mg, 47%) as a red solid.

### Synthesis of 6‐Biotin Aminohexanoic Acid

Biotin‐NHS ester (500 mg, 1.46 mmol) and 6‐aminohexanoic acid (384 mg, 2.93 mmol) were dissolved in DMF (10 mL) and cooled to 0 °C. TEA (1.02 mL, 7.32 mmol) was added, and the mixture was stirred at room temperature for 4 h. The product was detected by LCMS. The reaction was acidified to pH 6 with water (16 mL) and formic acid (2 mL), then refrigerated at 4 °C overnight. The precipitate was collected by filtration, washed with water (3 × 5 mL), and dried to yield 6‐biotin aminohexanoic acid (252 mg, 48%) as a white solid.

### Synthesis of Biotin‐Berberine

6‐Biotin aminohexanoic acid (100 mg, 0.28 mmol) was dissolved in DMF (2 mL), and HATU (160 mg, 0.42 mmol) was added at 0 °C. After stirring for 30 min, Berberrubine (100 mg, 0.28 mmol) and DIEA (217 mg, 1.68 mmol) were added, and the reaction was stirred overnight at room temperature. The product was detected by LCMS and purified using reversed‐phase flash chromatography (C18 silica gel column, MeCN in water with 0.05% HCl, gradient 5% to 50% over 30 min, UV detection at 254 nm). The crude product was further purified by preparative HPLC (Xselect CSH Prep Fluoro‐Phenyl C18 column, 30 × 150 mm, 5 µm; mobile phase A: water with 0.05% HCl, mobile phase B: ACN; flow rate: 60 mL min^−1^; gradient: 10% to 23% B over 10 min; detection at 254 nm/220 nm; retention time: 9.8 min). The final product, Biotin‐Berberine, was obtained as a white solid (5.4 mg, 2.7%). The synthesized Biotin‐Berberine was characterized by LCMS and ^1^H‐NMR (DMSO‐*d*_6_, 400 MHz; CD_3_OD, 400 MHz).

### Biotin Pulldown Assay

For the in vivo Biotin pulldown assay, HEK293T cells were treated with Biotin or Biotin‐labeled Berberine (10 µm, 2 h). Cells were lysed and harvested with RIPA buffer, containing 50 mm Tris‐HCl, 150 mm NaCl, 1% NP‐40, and 0.5% sodium deoxycholate. Balanced Streptavidin Agarose beads (ThermoFisher, Cat# 20357) were added and incubated overnight at 4 °C. The beads were washed with RIPA buffer adding 0.03% Tween 5 min for 5 times. The indicated proteins were detected by Immunoblotting.

For the in vitro biotin pulldown assay, biotin or biotin‐labeled berberine (2 µg) was pre‐incubated with either recombinant protein (2 µg) or in vitro transcribed/translated protein (Promega, Cat# L1170) for 8 h at 4 °C. The mixture was then incubated with streptavidin agarose beads (Thermo Fisher, Cat# 20357) overnight at 4 °C. The beads were washed as previously described and the bound proteins were analyzed by immunoblotting.

### Cell Thermal Shift Assay

Cells were collected and the pelleted cells were washed with PBS. Lysis was performed using RIPA buffer, followed by sonication for 15 s at 4 °C. The samples were then centrifuged at 12 000 g for 10 min at 4 °C. The protein supernatant obtained after centrifugation was treated with water (vehicle) or berberine at a concentration of 10 µm. After coincubation with the compounds for 2 h, the cells in each group were heated at different temperatures, ranging from 35 to 59 °C, for 30 min. Subsequently, the samples were centrifuged at 4 °C for 10 min, and the supernatants were analyzed by immunoblotting.

### Immunofluorescence and Confocal Microscopy

For visualizing the intracellular distribution of Berberine, HeLa cells were grown on coverslips, incubated with 50 nm MitoTracker DeepRed (ThermoFisher, Cat# M22426) in fresh medium for 15 min at 37 °C. Berberine (10 µm) was added for 10 min. After treatment, the medium was replaced with phenol red‐free DMEM supplemental with 10% FBS. To confirm that there was no spectral overlap between berberine and MitoTracker DeepRed, control experiments were performed where cells were stained with berberine or MitoTracker DeepRed individually. Berberine fluorescence was detected using the FITC filter (excitation: 488 nm, emission: 500–550 nm), while MitoTracker DeepRed fluorescence was detected using the Cy5 filter (excitation: 640 nm, emission: 655–700 nm). No signal crossover was observed between the two channels. For co‐localization analysis, cells stained with both berberine and MitoTracker DeepRed were visualized using a DeltaVision Deconvolution microscope (GE Healthcare). Images of berberine and MitoTracker DeepRed signals, acquired by FITC and Cy5 filters respectively, were merged to demonstrate their co‐localization in mitochondria.

For visualizing the intracellular distribution of Biotin‐labeled Berberine, HeLa cells were grown on coverslips, incubated with 10 µm Biotin‐Berberine for 10 min at 37 °C. After treatment, cells were fixed in 4% paraformaldehyde for 5 min. Next, the cells were incubated with 0.3% Triton X‐100 in PBS for 10 min on ice and then blocked in 3% BSA in PBS for at least 1 h. The primary antibody was incubated overnight at 4 °C followed by secondary antibody incubation. Finally, the cells were mounted using 80% glycerol. Antibodies were used at the following concentrations: anti‐COXIV, rabbit polyclonal, Cell Signaling Technology, Cat# 4850 (1:200); Alexa Fluor 488‐conjugated streptavidin antibody, ThermoFisher, Cat# S11223 (1:400); Alexa Fluor 546‐conjugated anti‐Rabbit Secondary Antibody, ThermoFisher, Cat# A11035 (1:400). Images were acquired using a DeltaVision Deconvolution microscope.

### Mitochondrial Isolation and Swelling Assay

For isolating cardiac mitochondria, mouse hearts were minced in mitochondrial isotonic buffer (225 mm mannitol, 75 mm sucrose, 5 mm MOPS, 0.5 mm EGTA, and 2 mm taurine, pH 7.25) supplemented with protease inhibitor cocktail, and subsequently homogenized using a dounce homogenizer. The homogenate was initially centrifuged for 10 min at 700 × g and the resulting supernatant was next spun for 10 min at 12 000 × g to pellet the mitochondria.

Mitochondrial swelling was measured as previously described.^[^ ^22^ ^]^ In brief, isolated cardiac mitochondria (100 µg) were resuspended in swelling buffer (120 mm KCl, 10 mm Tris‐HCl, 5 mm MOPS, 5 mm Na_2_HPO_4_, 10 mm glutamate, 2 mm malate, and 0.1 mm EGTA) in a total volume of 200 µl. Pore opening was induced and detected by the addition of 500 µm of total CaCl_2_ while monitoring absorbance at 540 nm. Where indicated, Berberine (10 µm), cyclosporin A (3 µm), or Ru360 (3 µm) was added.

### Isolation and Culture of Primary Neonatal Rate Cardiomyocytes (NRCMs)

NRCMs were isolated and cultured as described previously.^[^ ^37^ ^]^ Briefly, cardiomyocytes were isolated from the neonatal hearts of <24 h‐old Sprague–Dawley rats using PBS containing 0.03% trypsin and 0.04% collagenase type II. The digestion was halted by Dulbecco's modified Eagle's medium (DMEM), supplemented with 10% fetal bovine serum (FBS). After differential attachment for 90 min, the cells in suspension were then plated and cultured in DMEM with 10% FBS at 37 °C with 5% CO_2_ for 24 h.

### Hypoxia/Reoxygenation (H/R) Injury Model

The hypoxia/reoxygenation (H/R) injury of NRCMs or H9c2 cells was achieved in an incubator (Thermo Fisher Scientific, Madison, WI, USA) filled with 94% N_2_, 5% CO_2_, and 1% O_2_ in DMEM deprived of glucose and sodium pyruvate for 4 h, followed by 8 h‐reoxygenation by transferring the cells to a normoxia incubator with normal supplement of oxygen and glucose.

To examine the protective effect of BBR on H/R‐induced Ca^2+^ overload, NRCMs infected with Ad‐4mt‐GCaMP6 or H9c2 cells stained with Rhod2 were treated with BBR at a final concentration 10 µM throughout the H/R period. Then cells were visualized using a DeltaVision Deconvolution microscope (GE Healthcare). To assess mitochondrial Ca^2+^ level using Rhod2, cells labeled with Rhod2 were illuminated at 546 nm, and fluorescence was collected through a TRITC filter. To assess mitochondrial Ca^2+^ level using 4mt‐GCaMP6, cells labeled with 4mt‐GCaMP6 were illuminated at 488 and 390 nm, and fluorescence was collected through a FITC filter. Images were analyzed using Image J software. All images were background‐corrected by subtracting mean pixel values of a cell‐free region of interest.

### Statistics and Reproducibility

Statistical comparisons between only two groups were carried out using unpaired Student's *t*‐test or the Mann–Whiney *U*‐test when a normal distribution could not be assumed. For comparisons across multiple groups, a one‐way analysis of variance (ANOVA) was employed. Furthermore, when examining the effects of two independent variables or the interaction between them on a dependent variable, a two‐way ANOVA was utilized to discern any significant differences. Statistical calculations were carried out using GraphPad Prism 6.0. We tested data for normality and variance and considered a *P*‐value of less than 0.05 as significant. All experiments were performed three or more times independently under identical or similar conditions.

## Conflict of Interest

The authors declare no conflict of interest.

## Author Contributions

H.Z., S.C., N.C., and W.W. contributed equally to this work. X.P. conceived the study. H.Z., S.C., G.L., and H.Z. performed calcium measurements and biochemical experiments. H.Z., N.C., and J.G. contributed to mouse studies. H.Z. and W.W. performed Screening and Data Analysis. D.L. and L.Z. performed immunostaining and flow cytometry analysis. J.C., T.L., T.L., W.Z., Q.X., T.Z., A.‐L.L., X.‐M.Z., and X.P. contributed to interpreting the results. X.P. and A.‐L.L. supervised the research. X.P. and H.Z. wrote the paper.

## Supporting information



Supporting Information

Supporting Information

## Data Availability

The data that support the findings of this study are available from the corresponding author upon reasonable request.
