# Correction to “[Berberine is a Novel Mitochondrial Calcium Uniporter Inhibitor that Disrupts MCU‐EMRE Assembly]”

**DOI:** 10.1002/advs.202510152

**Published:** 2025-08-26

Haixin Zhao, Siqi Chen, Nian Cao, Wenjun Wu, Guangqin Liu, Jun Gao, Jiayi Chen, Ting Li, Dingyi Lu, Lingmin Zeng, Haizhen Zhu, Weina Zhang, Qing Xia, Teng Li, Tao Zhou, Xue‐Min Zhang, Ai‐Ling Li, and Xin Pan. Adv Sci (Weinh). 2025 May;12(17): e2412311.

https://doi.org/10.1002/advs.202412311

Description of the error:

In the originally published paper, Figure 1B contained an inadvertent duplication of representative images for cytosolic calcium and mitochondrial calcium levels at 90–180 min in the 10 µM berberine group. These images were mistakenly reused from the corresponding 5 µM berberine group due to an oversight during figure preparation. We hereby request a correction to Figure 1B as detailed below.

Corrected Figure 1B:


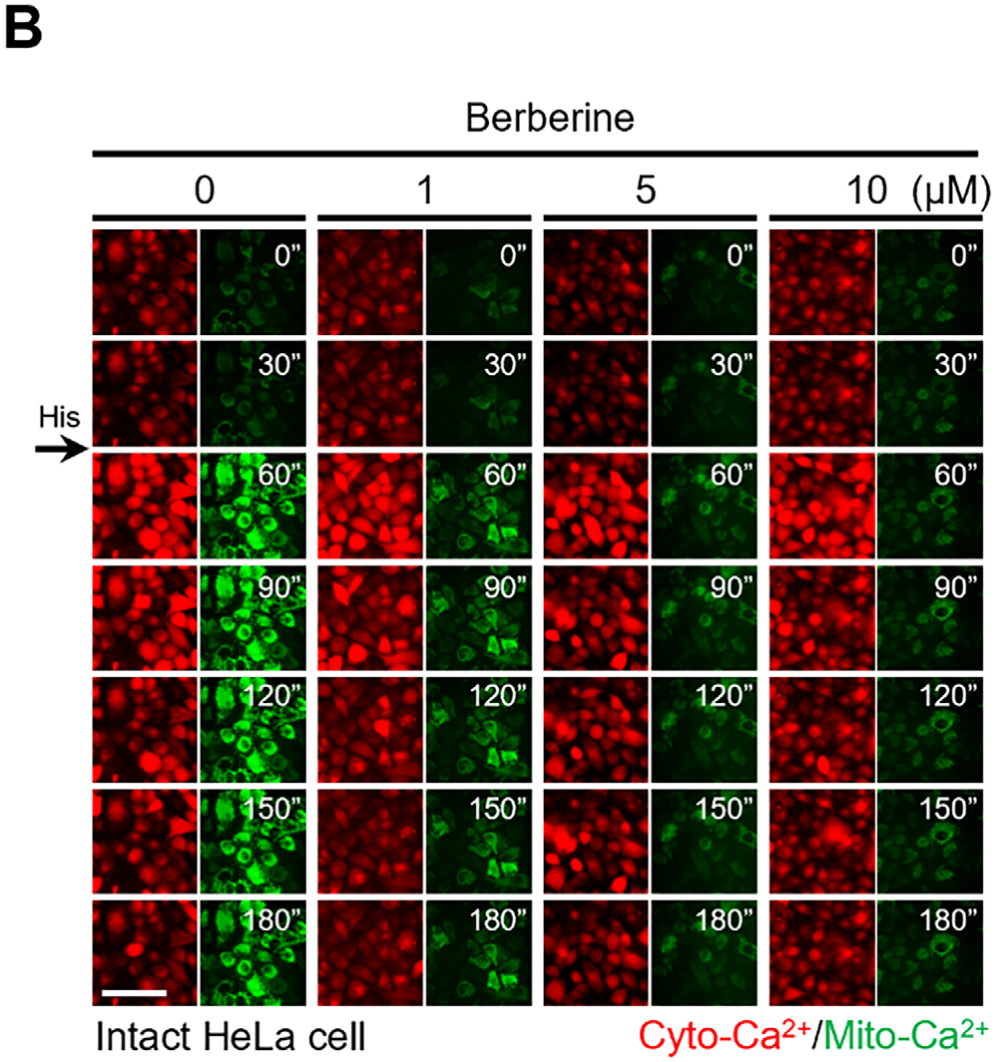


Additionally, we recognized that the images for mitochondrial calcium exhibited minimal changes between 60 and 180 min following histamine stimulation. This might have led readers to mistakenly assume that the fluorescence images at different time points were duplicated from the same data. To prevent any potential misunderstanding, we provided the original movies (Video V1–V4, Supporting Information) as supplementary material. Therefore, in Section 2.2 (page 2), the sentence: “…while simultaneously monitoring cytosolic and mitochondrial Ca^2^⁺ dynamics (Figure 1B).” should be updated to: “…while simultaneously monitoring cytosolic and mitochondrial Ca^2^⁺ dynamics (Figure 1B; Video V1–V4, Supporting Information).”

These corrections do not affect the conclusions of this paper. We apologize for these errors.

## Supporting information



Supplementary Video 1

Supplementary Video 2

Supplementary Video 3

Supplementary Video 4

