# Supplementary material for: Berberine is a Novel Mitochondrial Calcium Uniporter Inhibitor that Disrupts MCU‐EMRE Assembly
Source: Adv Sci (Weinh). 2025 Feb 7;12(17):2412311. doi: 10.1002/advs.202412311 (PMC12061237; doi:10.1002/advs.202412311)
Supplement: Supplementary file 1 — Supporting Information [file ADVS-12-2412311-s002.docx]

Supporting Information

Berberine is a novel mitochondrial calcium uniporter inhibitor that disrupts MCU-EMRE assembly

*Haixin Zhao, Siqi Chen, Nian Cao, Wenjun Wu, Guangqin Liu, Jun Gao, Jiayi Chen, Ting Li, Dingyi Lu, Lingmin Zeng, Haizhen Zhu, Weina Zhang, Qing Xia, Teng Li, Tao Zhou, Xue-Min Zhang, Ai-Ling Li*, and Xin Pan**

**Figure S1.**


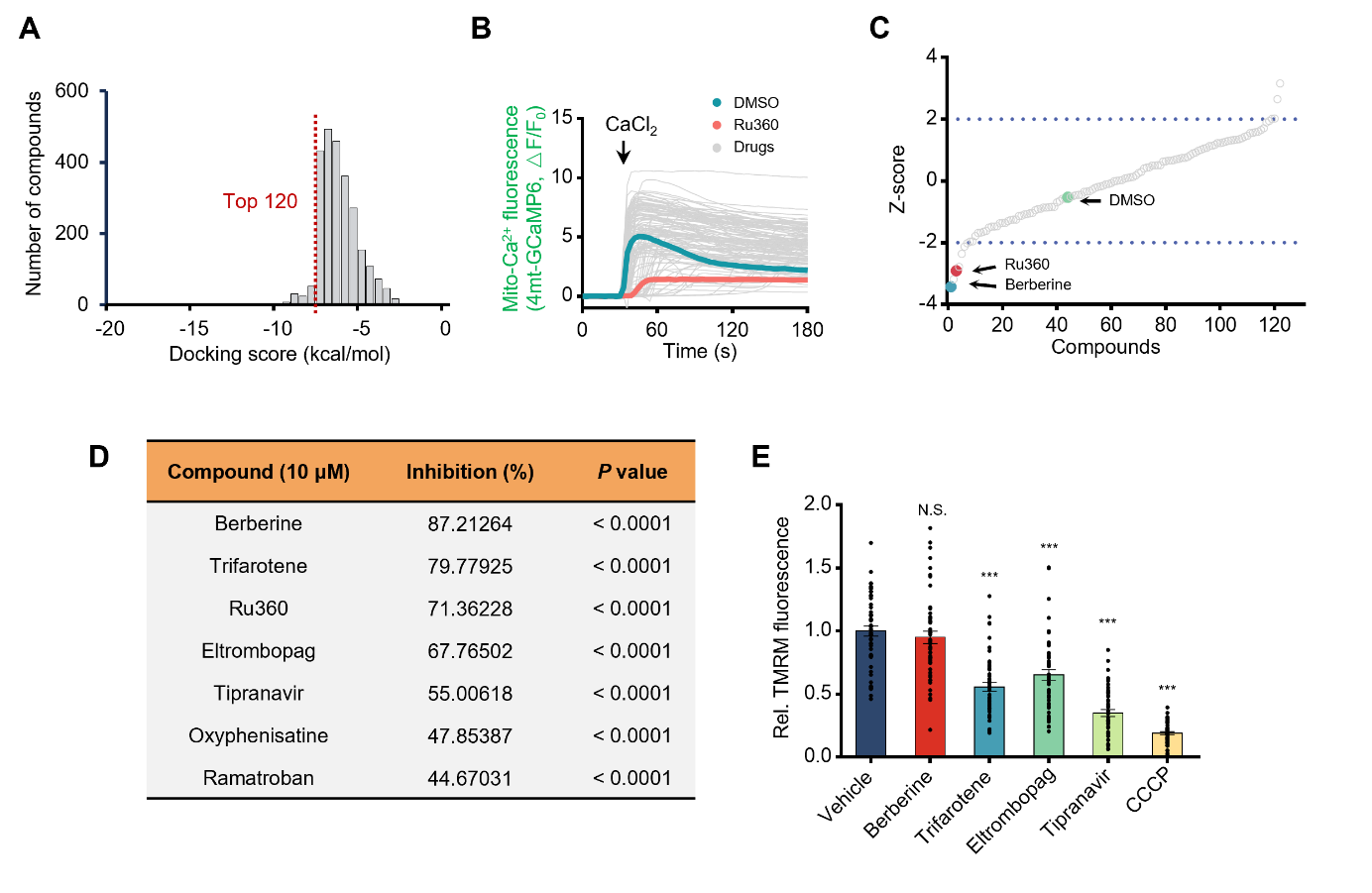


Figure S1. Screening identifies Berberine as an inhibitor of mitochondrial Ca^2+^ uptake. (A) Histogram plot of the 2,816 FDA-approved drug library based on the free-energy docking score with MCU. (B) Traces of mitochondrial Ca^2+^ level (indicated by fluorescence of 4mt-GCaMP6) in digitonin-permeabilized HeLa cells upon the addition of Ca^2+^ in the presence of DMSO, Ru360, or drugs. (C) Graphical representation of drug screen results. ‘Z-score’ ranks drugs based on maximal amplitudes of the traces in (B). (D) The list of the top 7 compounds with mitochondrial Ca^2+^ uptake inhibitory activity. (E) Measurement of mitochondrial membrane potential indicated by TMRM fluorescence intensity. Data are shown as the mean ± s.e.m. n = 50 cells for each condition. *P* value was analyzed using one-way ANOVA.

**Figure S2.**


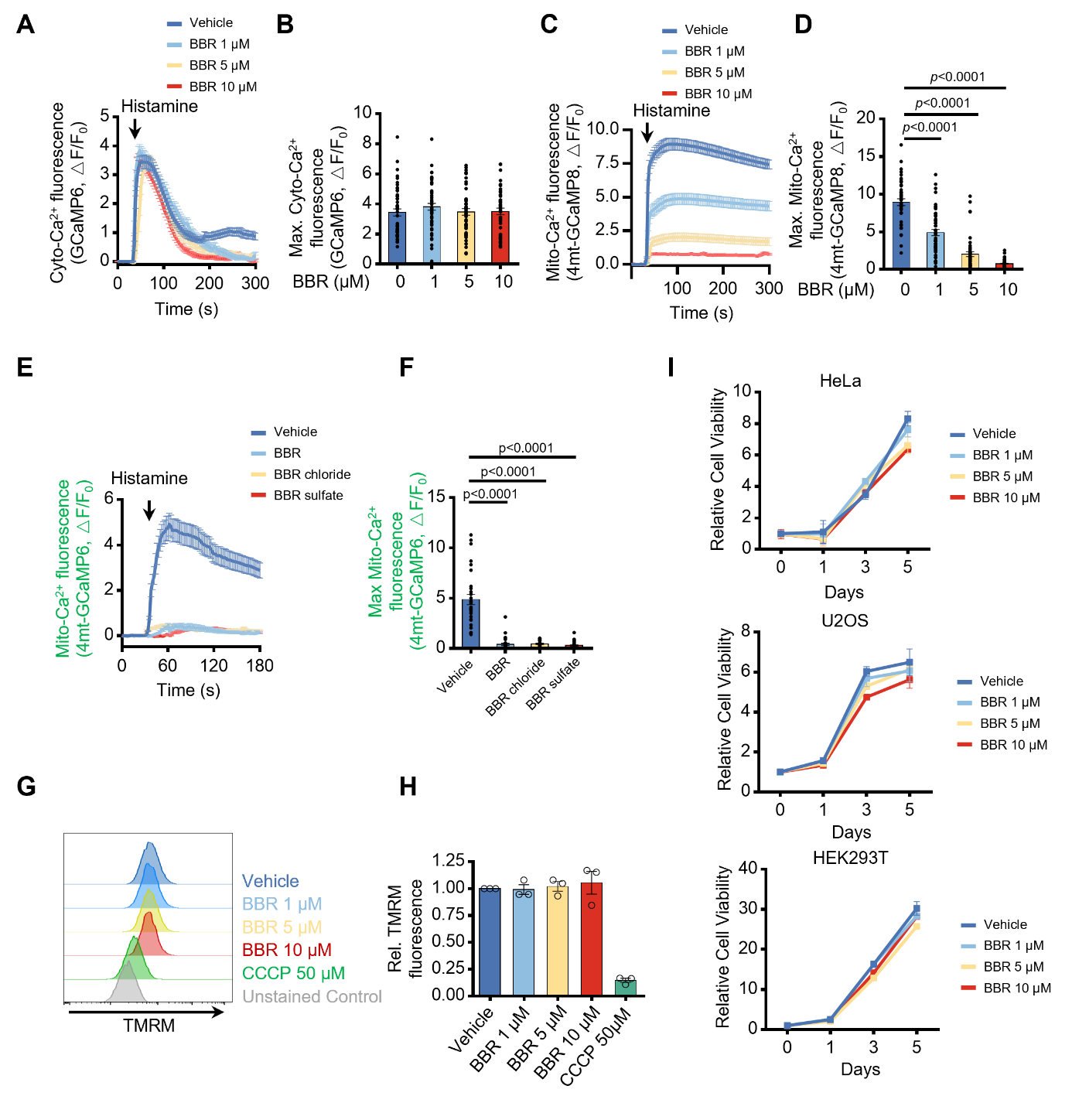


Figure S2. Berberine selectively inhibits mitochondrial Ca^2+^ uptake without impairing cytosolic Ca^2+^ signaling, mitochondrial membrane potential, or cell viability. (A) Representative traces of cytosolic Ca^2+^ level (indicated by GCaMP6) in 10 μM Histamine-stimulated HeLa cells pretreated with different concentration of Berberine. Data are shown as the mean ± s.e.m. n = 50 cells for each condition. (B) Measurement of the maximal amplitudes of the cytosolic Ca^2+^ traces in (A). Data are shown as the mean ± s.e.m. n = 50 cells for each condition. *P* value was analyzed using one-way ANOVA. (C) Representative traces of mitochondrial Ca^2+^ level (indicated by 4mt-GCaMP8) in 10 μM Histamine-stimulated HeLa cells pretreated with different concentration of Berberine. Data are shown as the mean ± s.e.m. n = 50 cells for each condition. (D) Measurement of the maximal amplitudes of the mitochondrial Ca^2+^ traces in (C). Data are shown as the mean ± s.e.m. n = 50 cells for each condition. *P* value was analyzed using one-way ANOVA. (E) Representative traces of mitochondrial Ca²⁺ levels (measured by 4mt-GCaMP6) in HeLa cells stimulated with 10 μM histamine and pretreated with 10 μM Berberine, Berberine chloride, or Berberine sulfate. Data are shown as the mean ± s.e.m. n = 30 cells for each condition. (F) Measurement of the maximal amplitudes of the mitochondrial Ca^2+^ traces in (E). Data are shown as the mean ± s.e.m. n = 30 cells for each condition. *P* value was analyzed using one-way ANOVA. (G) Flow cytometry analysis of TMRM staining in HeLa cells pretreated with different concentrations of Berberine and 50 μM CCCP. Shown is one experiment that is representative of three similar experiments. (H) Quantitative analysis of relative fluorescence of TMRM staining after different concentrations of Berberine treatment and 50 μM CCCP treatment. Data are shown as the mean ± s.e.m. n = 3 independent experiments. (I) Cell viability of HeLa cells, U2OS cells and HEK293T cells after different concentration of Berberine treatment was assessed at indicated time and normalized to day 0 in each group. Data are shown as the mean ± s.e.m. n = 3.

**Figure S3.**


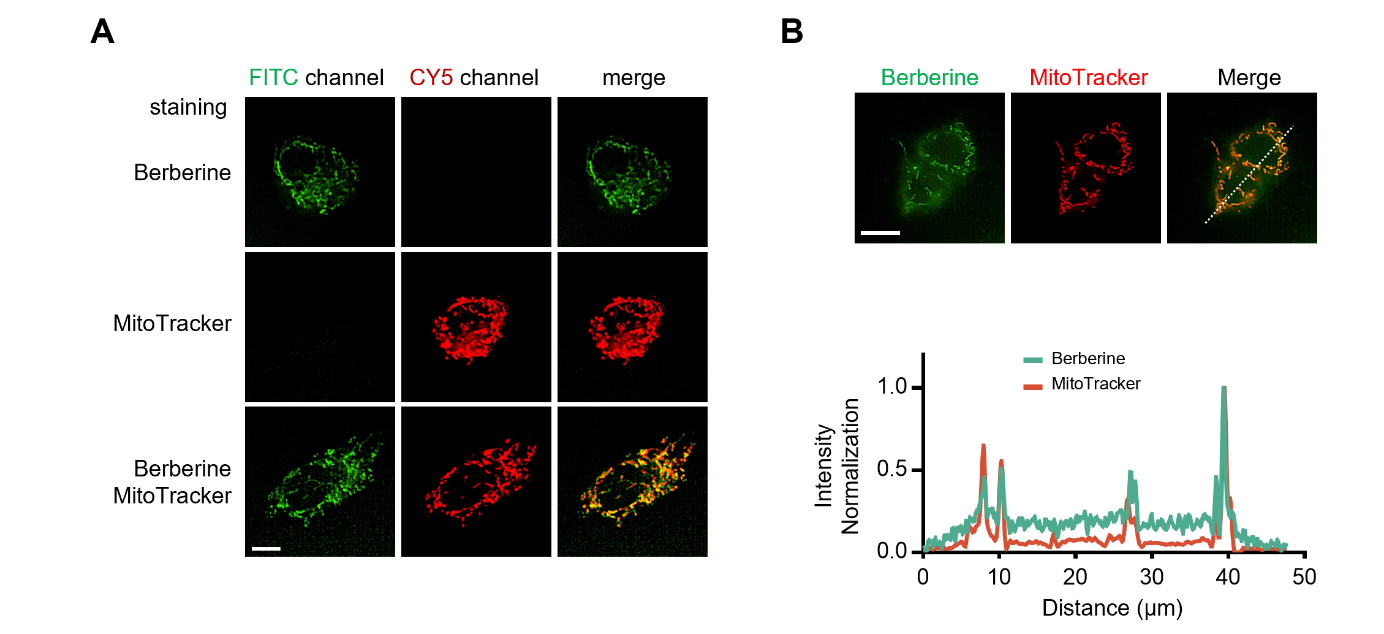


Figure S3. Berberine preferentially localizes to mitochondria. (A) Representative images of HeLa cells stained with Berberine or MitoTracker DeepRed, captured using FITC and CY5 channels. The merged image is shown in the right panel. (B) Representative images of HeLa cells co-stained with Berberine (green) and MitoTracker DeepRed (red). Normalized fluorescence intensity spatial profiles along the white line are shown below. Scale bar, 10 μm.

**Figure S4.**


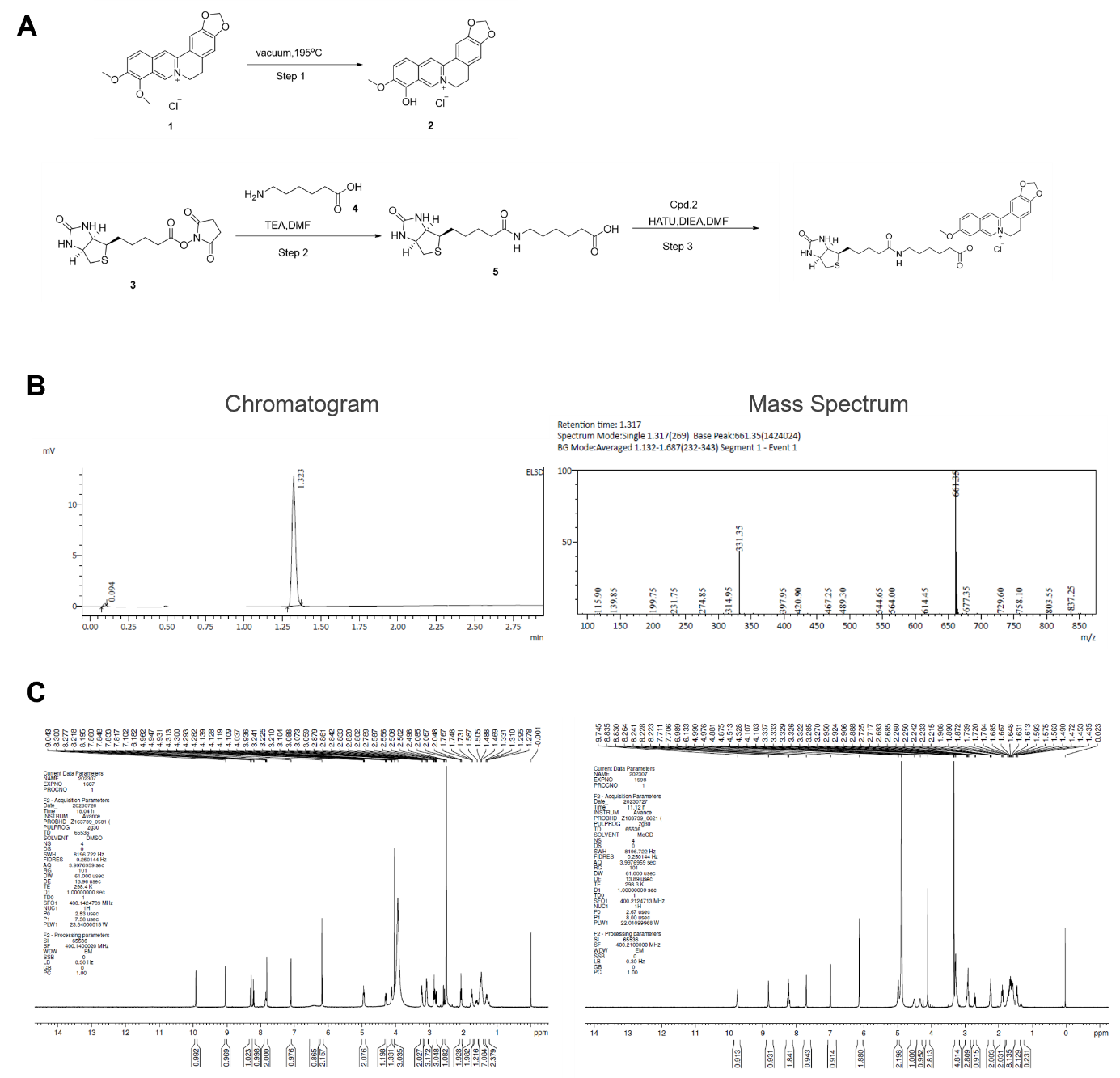


Figure S4. Synthesis and characterization of Biotin-Berberine. (A) Schematic diagram of the synthesis pathway for Biotin-Berberine. (B) Characterization of the synthesized Biotin-Berberine by LC-MS. (C) Characterization of the synthesized Biotin-Berberine by ^1^H-NMR (left: DMSO-*d*_6_, 400 MHz; right CD_3_OD, 400 MHz).

**Figure S5.**


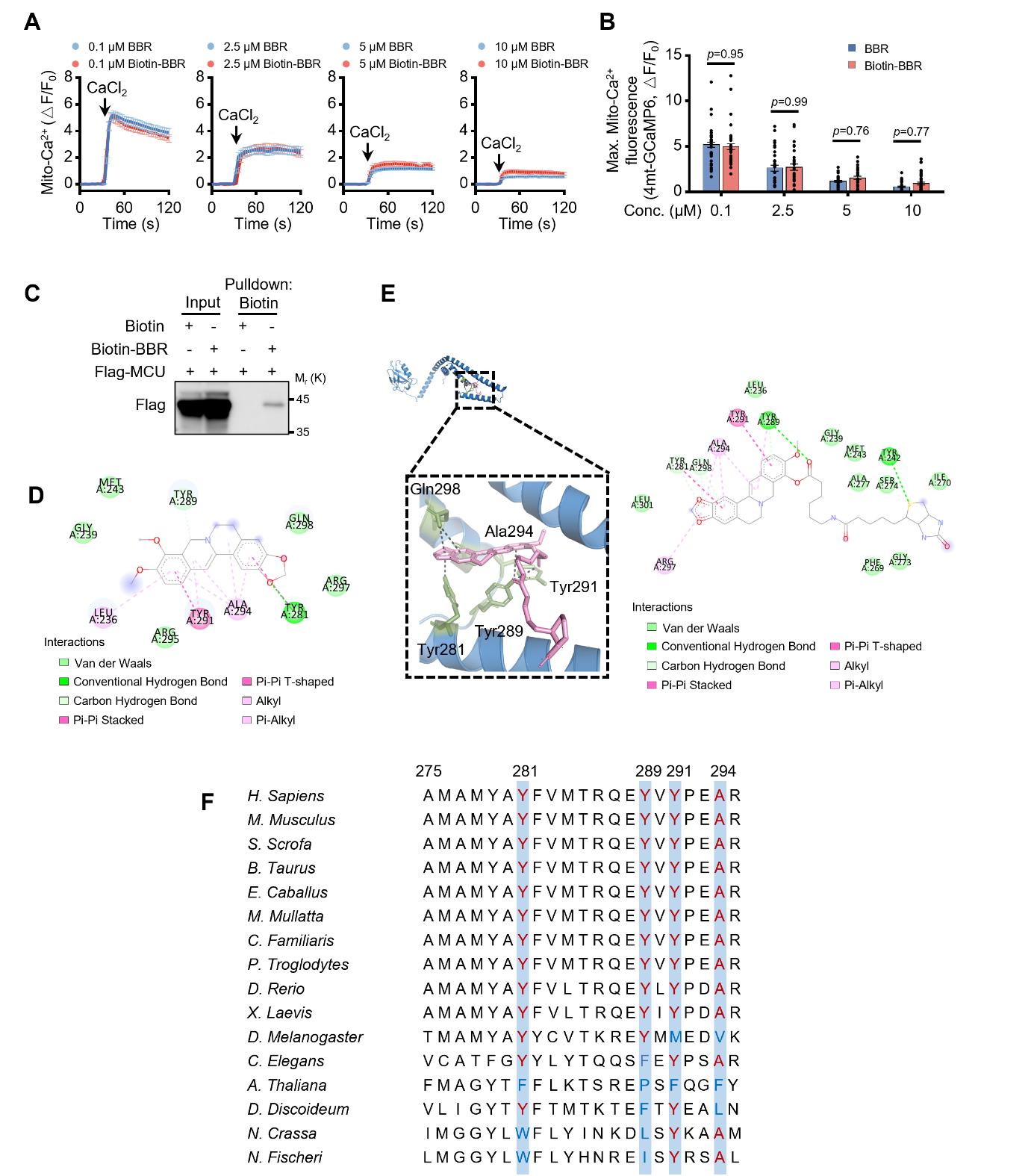


Figure S5. Berberine directly targets MCU. (A) Traces of mitochondrial Ca^2+^ level (indicated by fluorescence of 4mt-GCaMP6) in digitonin-permeabilized HeLa cells upon the addition of Ca^2+^ in the presence of different concentration of Berberine or Biotin-Berberine. Data are shown as the mean ± s.e.m. n = 40 cells for each condition. (B) Measurement of the maximal amplitudes of the mitochondrial Ca^2+^ traces in (A). Data are shown as the mean ± s.e.m. n = 40 cells for each condition. *P* value was analyzed using two-way ANOVA. (C) Western Blot analysis of Biotin-Berberine pulldown assay in MCU-Flag overexpressed HEK293T cells. Cells were treated with 2 h 10 μM Biotin or 10 μM Biotin-Berberine. (D) Two-dimensional diagrams illustrating the binding mode of berberine with MCU. (E) Molecular docking model of the interaction between human MCU and Biotin-Berberine. Two-dimensional diagrams on the right illustrate the binding mode of Biotin-Berberine with MCU. (F) Sequence alignment of the putative region (amino acids 275-295) surrounding the JML domain of MCU proteins from 16 diverse species.

**Figure S6.**


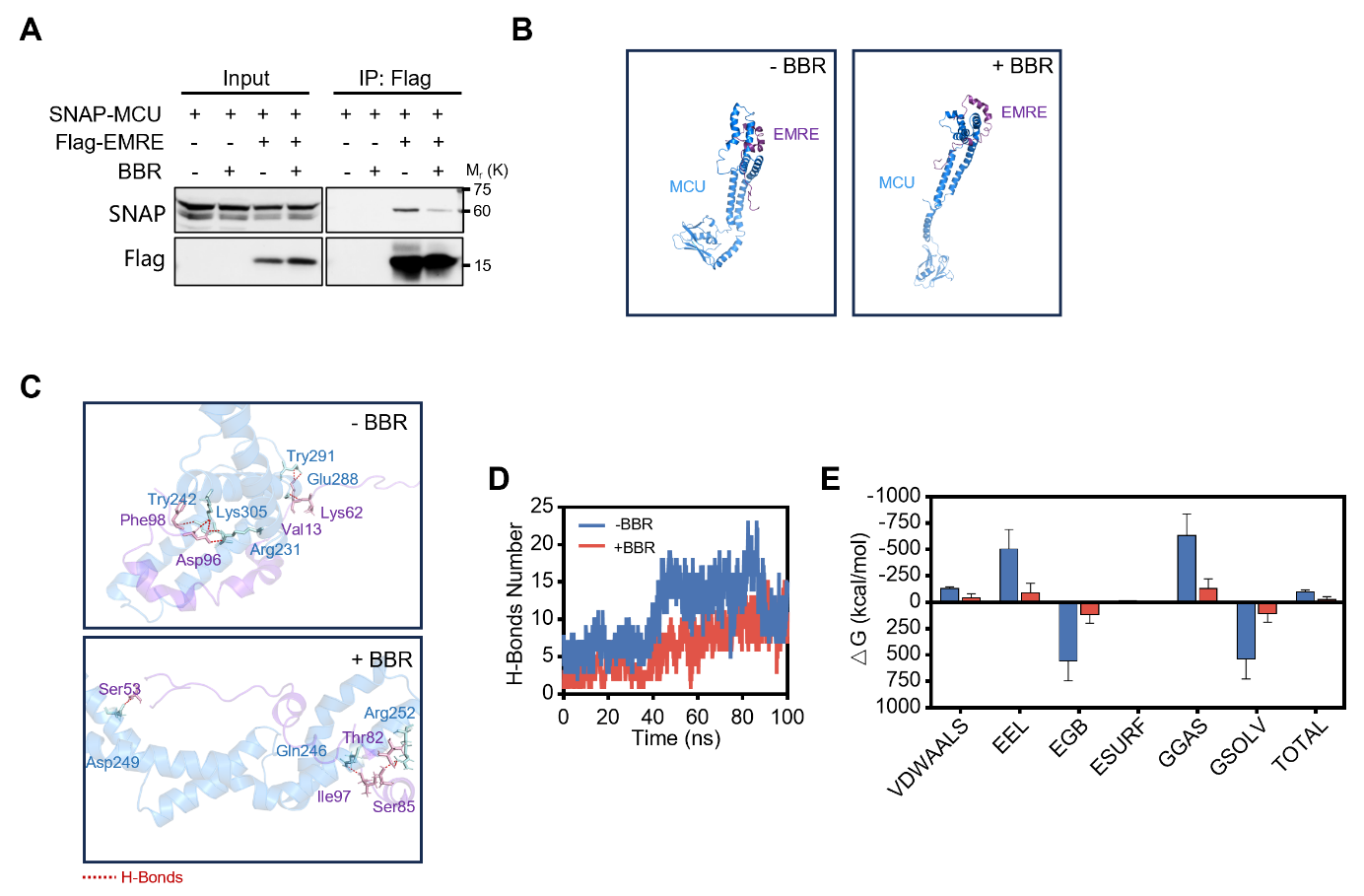


Figure S6. Berberine disrupts MCU-EMRE assembly. (A) Western Blot analysis of co-precipitation of the Flag-EMRE with SNAP-MCU in HEK293T cells in the presence of 10 μM Berberine or not for 2 hours. (B) Molecular dynamics simulations illustrating the assembly conformations of the MCU-EMRE complex in the presence and absence of Berberine. (C) Molecular dynamics simulations reveal the hydrogen bonding interactions between MCU and EMRE under conditions with and without Berberine. (D) Molecular dynamics simulations displaying the number of hydrogen bonds between MCU and EMRE in the presence and absence of Berberine. (E) Molecular dynamics simulations demonstrating the binding free energy between MCU and EMRE with and without Berberine. The components of the binding free energy are detailed as follows: van der Waals energy (VDWAALS), electrostatic energy (EEL), polar solvation energy (EGB), non-polar solvation energy (ESURF), total gas phase free energy (GGAS, calculated as the sum of VDWAALS and EEL), total solvation free energy (GSOLV, calculated as the sum of EGB and ESURF), and the total free energy (TOTAL, calculated as the sum of GSOLV and GGAS).

**Figure S7.**


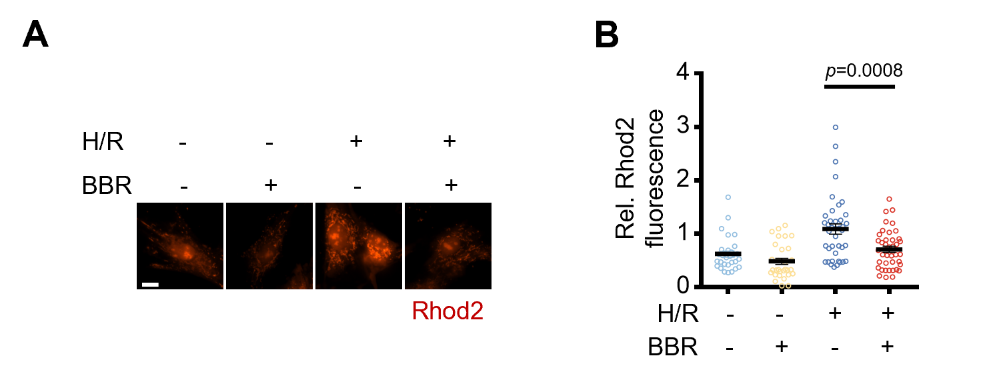


Figure S7. Berberine attenuates mitochondrial Ca^2+^ overload in cardiomyocytes under pathological conditions. (A-B) Measurement of mitochondrial Ca^2+^ level (indicated by Rhod2) in H9C2 cells. Cells were pretreated with 10 μM Berberine or not, and then were subjected to hypoxia (3 h) followed by reoxygenation (12 h) (H/R). Relative fluorescence intensity of Rhod2 were measured in (B). Data are shown as the mean ± s.e.m. n = 30 cells (Control and BBR groups), n = 40 cells (H/R and H/R+BBR groups). *P* value was analyzed using one-way ANOVA. Scale bar, 10 μm.
